# Human cancer evolution in the context of a human immune system in mice

**DOI:** 10.1002/1878-0261.12374

**Published:** 2018-09-03

**Authors:** Odd L. Gammelgaard, Mikkel G. Terp, Birgitte Preiss, Henrik J. Ditzel

**Affiliations:** ^1^ Department of Cancer and Inflammation Research Institute of Molecular Medicine University of Southern Denmark Odense Denmark; ^2^ Department of Pathology Odense University Hospital Denmark; ^3^ Department of Oncology Odense University Hospital Denmark

**Keywords:** adaptive resistance, cancer, humanized immune system mice, metastasis, programmed death ligand 1, tumor‐infiltrating lymphocytes

## Abstract

Immunotherapy is one of the most promising cancer treatment modalities, but the lack of appropriate preclinical *in vivo* models hampers the development of novel immunotherapeutic strategies. Here, we studied the ability of transplanted human cancer cells to form primary tumors and metastasize in humanized immune system (HIS) mice created by transfer of CD34+ human hematopoietic stem cells. All tested transplanted cancer cell lines developed primary tumors that progressed nearly synchronously. Spontaneous lung and liver metastases developed from both orthotopic and ectopic transplanted cancer cells, and the ability to spread inversely correlated with the extent of CD8+ infiltration in the primary tumor. Further analysis revealed that interactions between the cancer model and the tumor‐infiltrating lymphocytes created tumor microenvironments (TMEs) resembling clinical cancers. Some models were largely immune cell‐excluding, while others appeared to develop adaptive resistance to immune‐mediated destruction by increased expression of programmed death ligand 1 (PDL1) and recruitment of human regulatory T cells. Our data suggest that HIS mice may provide a promising *in vivo* tumor model for evaluating immune modulatory anticancer therapies. Moreover, our study identified different tumor models resembling specific types of human TMEs, rendering each beneficial for addressing disease‐specific issues.

**Abbreviations**
BRGS: BALB/c Rag2^−^/^−^ IL‐2Rγc^−^/^−^ SIRPα.NOD
EGFR: epidermal growth factor receptor
FoxP3: forkhead box protein P3
HIS: humanized immune system
HSC: hematopoietic stem cells
IHC: immunohistochemistry
PD1: programmed death 1
PDL1: programmed death ligand 1
TIL: tumor‐infiltrating lymphocyte
TME: tumor microenvironments
Tregs: regulatory T cells

## 1 Introduction

Metastases remain a major clinical challenge, accounting for more than 90% of cancer‐related deaths (Hanahan and Weinberg, 2011). Despite the time and resources invested in cancer therapy, the 5‐year survival rate of most metastatic cancers has improved only marginally, if at all, during the last decade (Steeg, 2016). While it is encouraging that immunotherapy, particularly immune checkpoint inhibitors, has recently improved overall survival of patients with certain types of cancer (Brahmer *et al*., 2012; Herbst *et al*., 2014; Hodi *et al*., 2010; Powles *et al*., 2014; Topalian *et al*., 2012), its effectiveness is limited to a small patient subpopulation. Thus, strategies to improve the anticancer immune response are being actively pursued.

Traditionally, human xenograft mouse models are used for translational cancer research and oncology drug development, but the immunodeficiencies of these conventional models restrict their use for development of immunotherapies and other treatments in which the interplay with the immune system influences the outcome. Alternative approaches include the use of syngeneic transplantable models as well as carcinogenic and genetically engineered mouse models wherein tumors develop in the context of a functional immune system. Unfortunately, however, human and mouse orthologous proteins often contain different epitopes, and even when cross‐species‐reactive antibodies or small molecules can be identified, the intracellular signaling and polarization of immune cells may differ (Gould *et al*., 2015), making proper translation challenging if not impossible. Murine cancer models are thus often unsuitable for evaluation of immune checkpoint inhibitors for human use, and it is essential to develop preclinical models in which human cancer development and metastasis can be investigated in the context of a functional human immune system.

To study and modulate human immune cells *in vivo*, different mouse models with variable humanized immune systems (HIS) have been developed, ranging from relatively simple models in which peripheral blood cells are transferred into immune‐deficient mice (PBL mice) to more advanced models in which human hematopoietic stem cells (HSC) are transplanted either alone or in combination with human lymphoid tissue for proper immune cell maturation (reviewed in ref. (Walsh *et al*., 2017)). The rapid onset of graft‐versus‐host‐like disease (Walsh *et al*., 2017) and the limited ability to mount primary immune responses currently limits the use of PBL mice for cancer studies. In HIS mice receiving HSC transplants (Lapidot *et al*., 1992; Legrand *et al*., 2011; Shultz *et al*., 2005, 2012; Traggiai *et al*., 2004), the HSCs engraft into the murine bone marrow and give rise to the major human immune cell subsets, including T, B, and dendritic cells (Legrand *et al*., 2006). Comparable to normal T‐cell development, immature double‐negative T cells home to the thymus, where they mature into double‐positive (Ishikawa *et al*., 2005), and subsequently single‐positive (CD4 or CD8) naïve (CD45RA+CCR7+) T cells, which seed secondary lymphoid organs (Legrand *et al*., 2011). Because the thymic cortical epithelial cells, normally the main mediators of positive selection (Fink and Bevan, 1978), are murine, the restriction of HIS mice‐matured T cells remains controversial. Nevertheless, mature T cells have been demonstrated to be restricted to the bone marrow MHC (HLA in HIS mice), but tolerant to the MHC of both species (H2 and HLA in HIS mice) in the xenogeneic setting (Zinkernagel and Althage, 1999; Zinkernagel *et al*., 1980). Furthermore, mature T cells from HIS mice have been shown to be functional and to engage in cytotoxic effector activities when exposed to allogeneic, but not syngeneic, HLA (Ishikawa *et al*., 2005; Traggiai *et al*., 2004). Despite these intriguing observations, very little has been reported regarding tumor development and the interactions with the immune system in HSC‐engrafted HIS mice. In addition to the technical difficulties and considerable cost of producing such mice, matching the HLA of cancer and HSC donors remains challenging. Intriguingly, it was very recently demonstrated that allogeneic tumors grow at a comparable pace in immune‐deficient and HIS mice. Furthermore, the authors showed that reconstituted immune cells were infiltrating transplanted tumors, and were able to delay tumor growth in a CD8+ T cell‐dependent manner when treated with a programmed death 1 (PD1) inhibitor (Wang *et al*., 2018). Building on these reports, we examined whether three well‐characterized human cancer cell lines [A375 (melanoma), and MDA‐MB‐231 and MDA‐MB‐468 (both triple‐negative breast cancer)] were able to develop primary tumors and distant metastasis in HIS mice. Additionally, we investigated whether, and how, the reconstituted immune system interacted with both primary tumors and metastases.

## 2 Materials and methods

### 2.1 Cells and animals

CD34+ HSC‐transplanted BALB/c Rag2^−^/^−^ IL‐2Rγc^−^/^−^ SIRPα.NOD (BRGS)‐HIS mice (Legrand *et al*., 2011) were purchased from Axenis S.A.S. (Fontenay‐aux‐Roses, France). Production data for each mouse are indicated in Table S1. Subconfluent A375, MDA‐MB‐231, and MDA‐MB‐468 cells were washed in PBS and harvested using EDTA (5 mm). Cells (1 × 10^6^) were resuspended in extracellular matrix from the Engelbreth‐Holm‐Swarm sarcoma. MDA‐MB‐231 (*n* = 5) and MDA‐MB‐468 (*n* = 5) were orthotopically transplanted into the R4 and L4 mammary gland of anesthetized female BRGS‐HIS mice, while A375 were transplanted subcutaneously in the right and left flanks of male BRGS‐HIS mice (*n* = 6). Primary tumor sizes were calculated using *V* = (W^2^ × *L*)/2. When primary tumors reached 1.2 cm in diameter, they were removed from anesthetized mice using blunt instrument dissection and collected for immunohistochemistry (IHC) analysis. Following a latency period to allow metastatic progression, all mice were euthanized and liver and lungs collected for IHC analysis. All animal experiments were approved by The Experimental Animal Committee, The Danish Ministry of Justice, and performed at the animal core facility of the University of Southern Denmark. The mice were housed under specific pathogen‐free conditions with *ad libitum* food and drinking water. The mice were euthanized if they showed any adverse signs or symptoms of disease, including weight loss, paralysis, or general discomfort.

### 2.2 Immunohistochemistry

Tissue sections from the formalin‐fixed and paraffin‐embedded tissue were cut, deparaffinized, and rehydrated prior to antigen retrieval by boiling in either Tris EGTA buffer (10 mm Tris and 0.5 mm EGTA, pH 9) for 15 min [for programmed death ligand 1 (PDL1 staining)], or in Cell Conditioning 1 buffer (Ventana Medical Systems, Oro Valley, AZ, USA) for 32 min (for CD3, CD8, CD4, CD45 staining) or 64 min [for forkhead box protein P3 (FoxP3) staining], or incubated with Protease 3 (Ventana Medical systems) at 36 °C for 4 min followed by 32 min of Cell Conditioning 1 buffer at 95 °C (for pan‐cytokeratin staining), or 8‐min treatment with protease 1 at 36 °C [Ventana Medical systems; for epidermal growth factor receptor (EGFR) staining]. Sections were incubated with anti‐CD3 (2GV6; Ventana Medical systems) for 8 min at 36 °C, anti‐CD4 (SP35; Ventana Medical systems) for 24 min at 36 °C, anti‐CD8 (1 : 100, M7103; DAKO, Glostrup, Denmark) for 32 min at 36 °C, anti‐CD45 (2B11&PD7/26; Ventana Medical systems) for 32 min at 36 °C, anti‐FoxP3 (1 : 40, 236A/E7; ThermoFisher Scientific, Waltham, MA, USA) for 16 min at 36 °C, anti‐PDL1 (1 : 500, EPR19759; Abcam plc., Cambridge, UK) for 1 h min at room temperature, anti‐EGFR (3C6; Ventana Medical systems) for 12 min at 36 °C or anti‐pan‐cytokeratin (1 : 30, KL1; AbD Serotec, Hercules, CA, USA) for 1 h at room temperature. Primary antibody binding was detected with either OptiView DAB IHC detection kit (760–700; Ventana Medical systems; CD3, CD4, CD8, CD45, pan‐cytokeratin, EGFR, FoxP3) or Envision FLEX DAB (DAKO; PDL1) as chromogen. All sections were counterstained with hematoxylin. Hematoxylin and eosin staining was performed by routine stainings. Slides were scanned at ×20 magnification using nanozoomer 2.0‐HT Whole Slide Imager (Hamamatsu, San Diego, CA, USA).

### 2.3 Quantification

Scanned slides were divided into sections using ndp.view 2.3.14 software (Hamamatsu) and subsequently semiquantified using imagej software as described previously (OpenWetWare, 2012). For each marker, semiquantified slides were normalized to manually counted areas of at least 1 mm^2^. At least 6 mm^2^ or 100% of the viable tumor tissues were analyzed.

### 2.4 Flow cytometry

Precancer transplantation blood analysis was performed by Axenis S.A.S on peripheral blood harvested from facial or retro‐orbital vein puncture in EDTA‐coated microtubes. Upon Ficoll density purification, unspecific binding was blocked by human and murine Fc‐block reagents. Leukocytes were subsequently stained with a cocktail of anti‐hCD3 (E450, UCH1, eBioscience, San Diego, CA, USA), anti‐hCD14 (FITC, 18D11; Immunotools, Friessoythe, Germany), anti‐hCD19 (PE, HIB19; BD, Franklin Lakes, NJ, USA) anti‐hCD11c (PE‐Cy7, Bu15; BioLegend, San Diego, CA, USA), anti‐hCD33 (APC, WM53; BD), anti‐hCD56 (PerCP E710, CMSSB; eBioscience), anti‐hCD45 (AF700, H130; BioLegend), and anti‐mCD45 (APC E780, 104; eBioscience). All data acquisitions were performed using a LSR‐II Fortessa flow cytometer. Data analysis was performed using flowjo (Treestar, Inc., Ashland, OR, USA) and graphpad prism‐5 software (GraphPad Software, Inc., La Jolla, CA, USA).

### 2.5 Statistical analysis

Statistical analyses were performed using prism 7.0b (GraphPad Software) and stata/ic 15.0 (StataCorp LLC, College Station, TX, USA). Immune cell infiltrates between cancer models were compared using Student's t‐test, and analysis of immune cell infiltrates between anatomical positions and tumor models where performed by multilevel mixed‐effects regression on averaged immune cell densities from manually counted areas. A *P* value of ≤ 0.05 was considered significant.

## 3 Results

### 3.1 Primary tumor growth and metastasis of human cancers in humanized immune system mice

**Figure 1:**
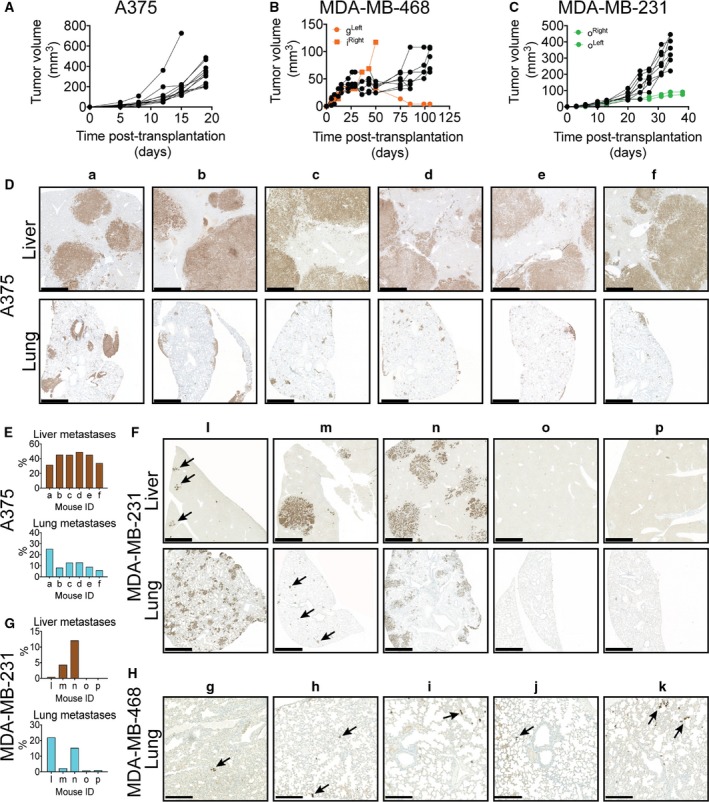
Growth and metastasis formation in HIS mice. Tumors from the cell lines (A) A375 (*n* = 6 mice), (B) MDA‐MB‐468 (*n* = 5 mice), and (C) MDA‐MB‐231 (*n* = 5 mice) all expanded despite the presence of an allogeneic immune system. Primary tumors derived from A375 consistently yielded multiple large liver and lung metastases (D,E), which was only occasionally observed in mice carrying MDA‐MB‐231‐derived primary tumors (F,G). Few cancer cells were detected in the lungs of MDA‐MB‐468‐transplanted mice (image showing the most densely cancer cell‐containing areas) (H) and were absent in the liver. Cancer cells were detected by IHC staining using antibodies against human EGFR (A375) or pan‐cytokeratin (MDA‐MB‐231 and MDA‐MB‐468). Scale bar 1 mm (D,F) and 250 μm (H), respectively.

After orthotopic or ectopic transplantation of cancer cells, tumors developed on both left and right sides of all HIS mice transplanted with the two human triple‐negative breast cancer cell lines, MDA‐MB‐231 (5/5 mice) and MDA‐MB‐468 (5/5 mice), and the human melanoma cell line A375 (6/6 mice), demonstrating that the presence of the allogeneic human immune system did not prohibit primary tumor development. In the mice transplanted with A375 cells, rapid, close‐to‐synchronous tumor growth was observed in 11 of 12 tumors (Fig. 1A), while the MDA‐MB‐468 cell line consistently developed only small tumors, reaching a volume of ~ 50–100 mm^3^ after 15 weeks (Fig. 1B). In mice transplanted with MDA‐MB‐231 cells, eight of 10 tumors grew uniformly (Fig. 1C). Interestingly, two tumors from the same mouse (mouse o) grew considerably slower than MDA‐MB‐231 tumors from other mice.

Having established that primary tumors developed and expanded relatively synchronously within each tumor model, we next evaluated whether the ability to metastasize was retained in the HIS mouse model. When primary tumors reached the maximum legal size of 1.2 cm, they were surgically removed and mice were followed to allow evaluation of potential development of metastases. Since melanoma often metastasizes to the brain, lung, and liver, we investigated the presence of A375 cells in these tissues by IHC. Multiple and very large liver and lung metastases were detected in all mice carrying A375 primary tumors (Figs 1D and S1), comprising 31–49% and 6–26% of the liver and lung area, respectively, in the analyzed sections (Fig. 1E). Brain metastases were not detectable in any mice (Fig. S2). In mice bearing luciferase‐transduced MDA‐MB‐231 and MDA‐MB‐468 tumors, metastasis development was monitored by intravital imaging (Fig. S3) and subsequently confirmed by IHC at endpoint. Macroscopic liver and lung metastases were observed in three MDA‐MB‐231 mice (mouse l, m, and n), but they varied greatly in both size and number (Figs 1F–G and S1). In mouse o, no lung or liver metastases were detected, whereas mouse p developed a few very small liver metastases, but no lung metastases. The MDA‐MB‐468‐transplanted mice consistently presented with small groups of single cancer cells in the lungs (Fig. 1H), but no sign of liver metastases or single cancer cells in the liver. Together, these data demonstrate that spontaneous metastases can develop from both ectopic and orthotopic primary tumors in HIS mice.

### 3.2 Tumor‐infiltrating immune cells in HIS mice: location, type, and extent

**Figure 2:**
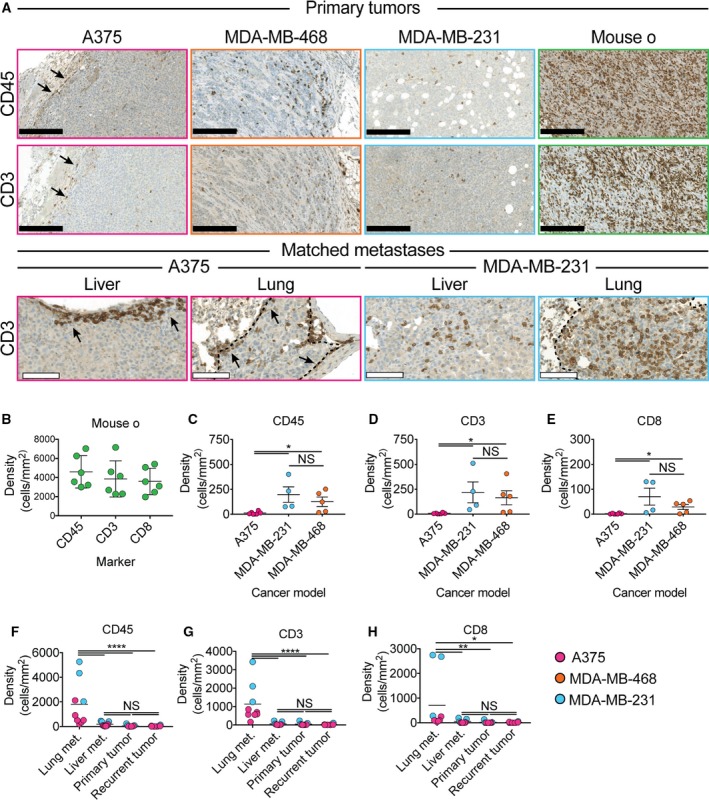
Type and location of tumor‐infiltrating immune cells. Tumors derived from the A375 cell line were largely immune cell‐excluding, whereas tumors derived from the MDA‐MB‐468 and MDA‐MB‐231 cell lines were highly infiltrated by CD45+, CD3+, and CD8+ cells (A–E). Although the position of immune cells within metastases was largely similar to the matched primary tumor, lung metastases derived from both A375 and MDA‐MB‐231 were significantly more densely infiltrated by CD45+ (F), CD3+ (G) and CD8+ (H) than matched primary tumors, liver metastases and recurrent tumors (*, *P *< 0.05 by Student's *t*‐test, C–E *n* = 4–6, F‐H *n* = 6–9). Black and white scale bar 250 μm and 100 μm, respectively.

Because location, type, and density of infiltrating immune cells are known to influence clinical outcome of several types of cancer (Fridman *et al*., 2012), we analyzed these factors in tumors from humanized mice. A375 primary tumors and metastases were poorly infiltrated by both CD3+ and CD45+ cells, and the few detectable cells were largely restricted to the tumor border and surrounding stroma (Fig. 2A). In striking contrast, both CD45+ and CD3+ cells were detected throughout the viable parts of MDA‐MB‐468 and MDA‐MB‐231 primary tumors and MDA‐MB‐231 metastases, albeit with some heterogeneity (Fig. 2A). Interestingly, the tumors from mouse o that grew considerably slower than other MDA‐MB‐231 tumors were unusually densely infiltrated by CD45+, CD3+, CD4+, and CD8+ cells. Indeed, the extent of infiltrating CD45, CD3, and CD8 cells in tumors from mouse o was statistically significant outliers compared to other MDA‐MB‐231 tumors (*P *< 0.01, Grubbs' test) and therefore analyzed separately (Figs 2A,B and S4). Based on IHC sections, we estimated the density of CD45+, CD3+, and CD8+ cells within all tumors, and confirmed that A375 tumors were significantly less densely infiltrated than MDA‐MB‐468 or MDA‐MB‐231 tumors, which, with exception of tumors from mouse o, were infiltrated to similar extents (Fig. 2C–E). Quantification further revealed that within all tumor models, CD3+ tumor‐infiltrating lymphocytes (TILs) comprised the major immune cell population (> 50%), although the B‐cell compartment was most efficiently reconstituted (Table S1), indicating that TILs enter the tumor bed as an active process. Interestingly, other types of immune cells, including plasma cells, neutrophils, and eosinophils, were also present (Fig. S5). In all models, the TILs were predominantly CD4+, with CD8+ cells accounting for 20–30%. Comparing matched primary tumor and metastatic lesions revealed a significantly higher density of CD45+, CD3+, and CD8+ cell infiltration in lung metastases compared to primary tumors and liver metastases in mice transplanted with both A375 (6/6 mice) and MDA‐MB‐231 cells (3/3 mice; Fig. 2F–H), indicating that lung metastasis is particularly accessible for immune cells in HIS mice.

Taken together, these data demonstrate that the investigated tumor models allow different levels of immune cells, and in particular lymphocytes, to access the tumor bed. The data further suggest that the accessibility of tumors is not only dependent on tumor intrinsic factors, but also their anatomic position.

### 3.3 The density of TILs in primary tumors inversely correlates with the capacity to form lung metastases

**Figure 3:**
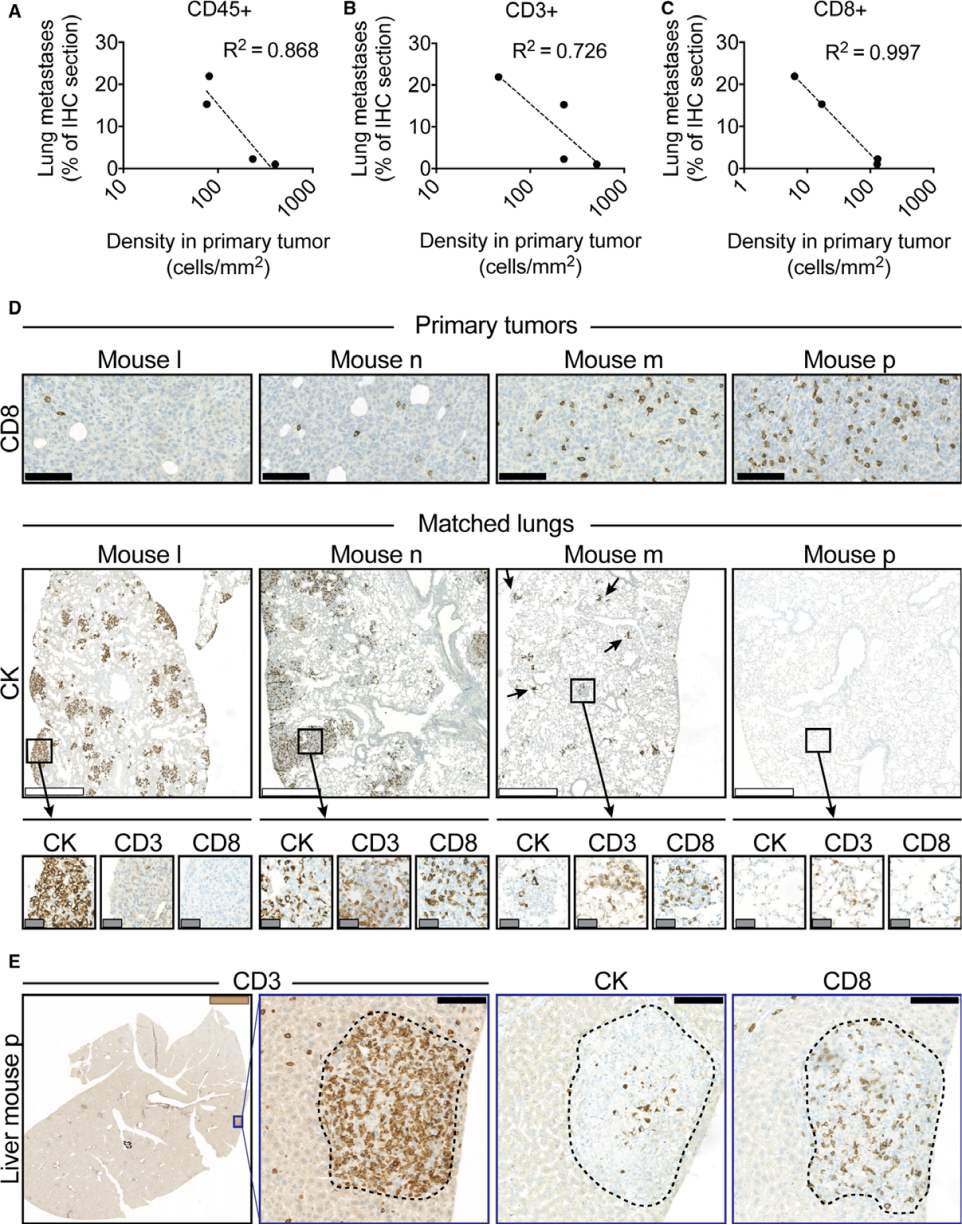
TIL density strongly correlates with the capacity to develop MDA‐MB‐231 lung metastases. In mice with disseminated MDA‐MB‐231 cancer, the amount of lung metastases inversely correlated to the amount of infiltrating (A) CD45+ (*R* ^2^ = 0.0.868), (B) CD3+ (*R* ^2^ = 0.0.726), and (C) CD8+ (*R* ^2^ = 0.997) cells in the primary tumor. The tumor cell/TIL ratio within lung metastases also reflected the density of immune cells within the matched primary tumor (D). The mouse with the most intense immune infiltration in its primary MDA‐MB‐231 tumor (mouse p) and disseminated disease did not present with any detectable lung metastases, but small liver metastases that were extensively infiltrated by TILs (E). Black, white gray, and brown scale bar 100 μm, 500 μm 50 μm, and 2.5 mm, respectively.

Having established that MDA‐MB‐231 tumors were being infiltrated by human immune cells, and that MDA‐MB‐231 could form distant metastasis, we investigated whether the rather variable extent of metastasis formation correlated with the density of immune cell subsets in the primary tumors. While the extent of liver metastasis appeared to occur independent of the immune system, we discovered an inverse correlation between the density of CD45+ (*R* ^2^ = 0.868), CD3+ (*R* ^2^ = 0.726), and especially CD8+ cell (*R* ^2^ = 0.997) in the primary tumor and the extent of lung MDA‐MB‐231 lung metastases (Fig. 3A–C). Further analysis of the lungs revealed that not only the extent of lung metastasis, but also the tumor cell/TIL ratio in the lung metastases, correlated with the immune infiltration in the primary tumor (Fig. 3D). Considering that lung metastasis appeared to be more accessible to immune cells than liver metastasis (Fig 2F–H), it is noteworthy that mouse p (most densely infiltrated by TILs in the primary tumor) did not show lung metastasis, but many small liver metastasis that were surrounded and greatly outnumbered by CD3+ and, to a lesser extent, CD8+ TILs (Fig. 3E). Combined with the observation that the only completely metastasis‐free MDA‐MB‐231 mouse (mouse o) presented with extremely infiltrated primary tumors that displayed inhibited growth (Figs 1C–G and 2B,C), our data strongly suggest that both primary tumor growth and metastasis formation can be controlled by the immune system in BRGS‐HIS mice. By extension, these data suggest that the reconstituted immune systems retain the effector function activities necessary for mounting effective anticancer immune responses. Finally, our results suggest that the seeding and outgrowth of metastatic lesions are considerably more sensitive to the presence of the human immune system than the primary tumors.

### 3.4 Common immune escape mechanisms are recapitulated in the HIS mouse model

**Figure 4:**
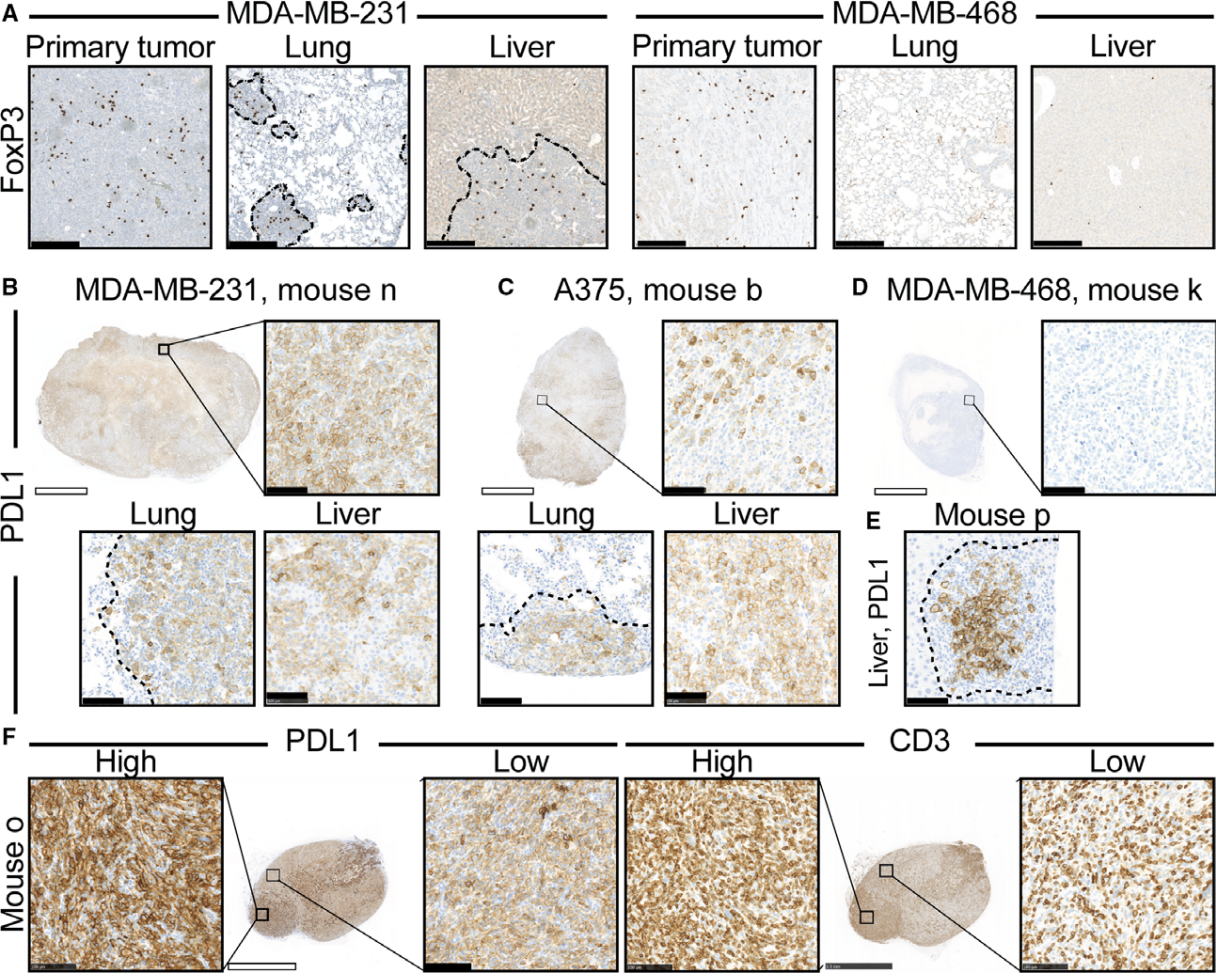
Common immune escape mechanisms, such as recruitment of Tregs and PDL1 expression by immune and cancer cells, are observed in the HIS model. MDA‐MB‐231 and MDA‐MB‐468 tumors consistently presented with high‐density Treg infiltration (FoxP3+), whereas Tregs were absent in healthy parenchyma (A). MDA‐MB‐231 and A375 tumors expressed PDL1 (B,C), whereas MDA‐MB‐468 tumors did not (D). MDA‐MB‐231 metastases contained higher numbers of PDL1+ cells than cancer cells, indicating PDL1+ immune cell recruitment (E). Within MDA‐MB‐231 tumors, PDL1 expression was increased in areas with high TIL infiltration (F). Dotted lines indicate tumor borders. White and black scale bars denote 2.5 mm and 100 μm, respectively.

Recruitment of Tregs is one of the best‐understood mechanisms by which cancers evade immune destruction. Because Treg infiltration in both melanoma (Miracco *et al*., 2007; Tefany *et al*., 1991) and breast cancer (Bates *et al*., 2006; Gobert *et al*., 2009) has been associated with poor prognosis, we investigated whether any of the applied cancer models in BRGS‐HIS mice recruited Tregs (FoxP3+) cells. Indeed, MDA‐MB‐231 and MDA‐MB‐468 primary tumors, and the metastatic lesions of MDA‐MB‐231, contained high densities of FoxP3+ cells that, in contrast, were largely absent in normal lung and liver parenchyma (Fig. 4A). In contrast, A375 primary tumors and metastatic sites were largely free from FoxP3+ cells (Fig. S6). Thus, it appears that certain cancer models are able to recruit Tregs in the BRGS‐HIS mouse model.

Inhibition of type I immune responses by expression of PDL1 either on cancer or recruited immune cells is another established immune escape mechanism. MDA‐MB‐231 and A375 primary tumors and metastatic sites consistently expressed PDL1, although generally more homogenously on MDA‐MB‐231 cancer cells (Fig. 4B,C). In striking contrast, PDL1 was almost undetectable in MDA‐MB‐468 primary tumors (Fig. 4D). Interestingly, comparisons of matched stainings of cytokeratin (Fig. 3E) and PDL1 (Fig. 4E) suggest that not only cancer cells, but also recruited cells within MDA‐MB‐231 tumor sites, express PDL1. Within MDA‐MB‐231 tumors, PDL1 expression was strongest in tumors densely infiltrated by TILs (compare Fig. 4B with F). Further, within tumors, the most TIL‐dense areas significantly overlapped with increased PDL1 expression (Fig. 4F). Taken together, these data imply MDA‐MB‐231 tumors recruit PDL1+ immune cells and increase PDL1 expression in areas with high TIL density, whereas A375 express PDL1 independently of TIL infiltration.

### 3.5 Pretransplantation blood analysis has limited predictive value of anticancer immune response

**Figure 5:**
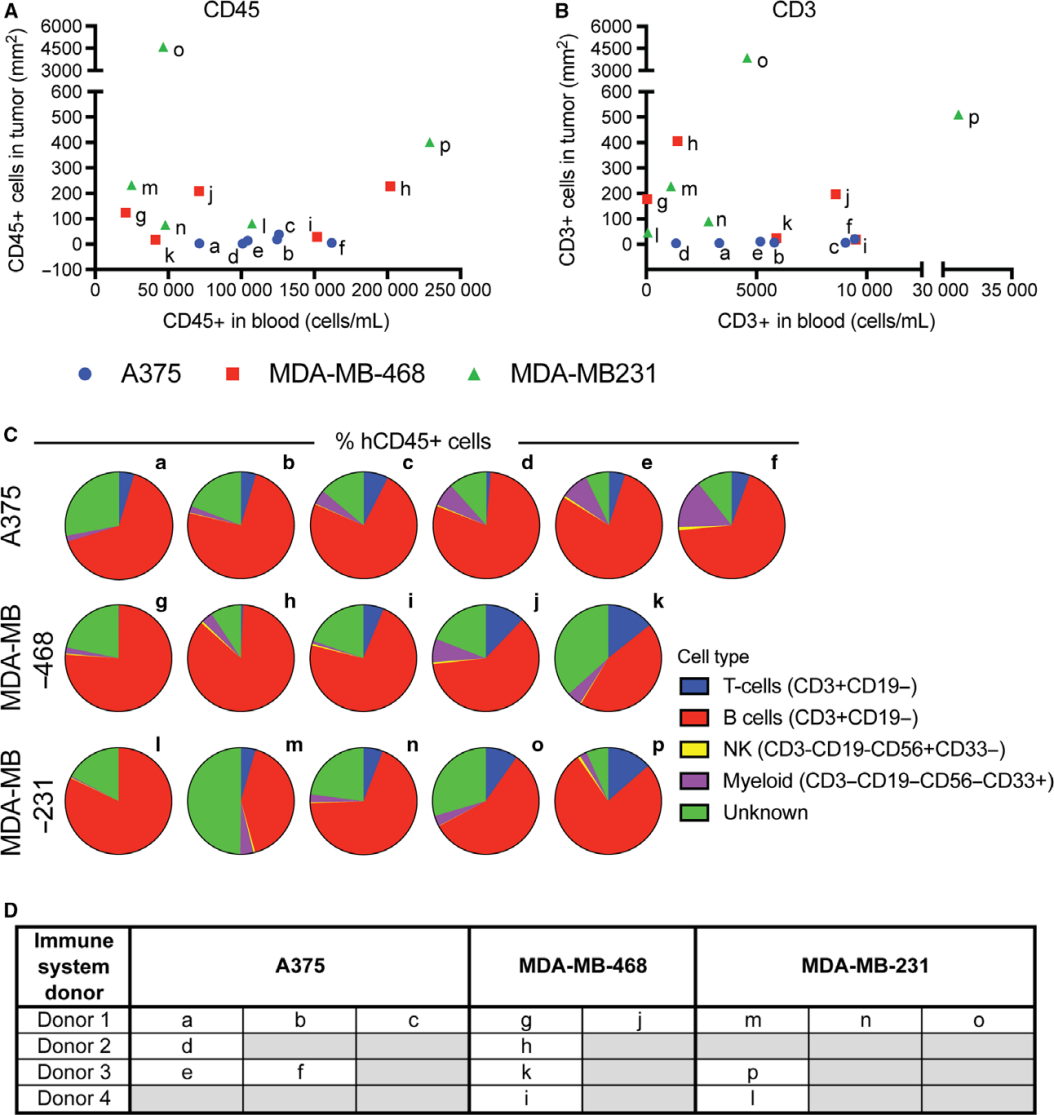
Pretransplantation immune cell frequency does not predict the extent of TILs. No correlation between the frequency of CD45+ (A) and CD3+ (B) cells in peripheral blood pretransplantation and the density of the same cells within A375, MDA‐MB‐468 and MDA‐MB‐231 tumors was found. No distinct pattern in the distribution of T, B, NK, or myeloid cells was observed in mice transplanted with the three cancer models (C). No obvious link between the specific HSC donor use and TIL frequency could be identified (D).

In addition to the anticipated intermodel variations in TIL frequency, considerable variations between mice, particularly the MDA‐MB‐468‐ and MDA‐MB‐231‐transplanted mice, was observed (Fig. 2B–E). These variations were not generally reflected by the amount of circulating human immune cells (CD45+) or lymphocytes (CD3+) in the mice prior to transplantation (Fig. 5A,B), although the surprisingly low TIL frequency in the tumors of mouse l may be explained by the low frequency of CD3+ cells in the blood due to poor human immune cell reconstitution. On the other hand, very few CD3+ cells were detected in the blood of mice m, g, and h, although tumors from these mice were intermediately infiltrated by CD3 cells (Fig. 5B), indicating that a low frequency of circulating CD3+ cells does not predict a low anticancer response. Conversely, mice c, f, and i contained relatively high levels of circulating CD3+ cells, yet their tumors were poorly infiltrated, indicating that high levels of circulating CD3+ cells are not sufficient to predict an immune response even in the allogeneic setting. Further, mouse o contained intermediate levels of circulating CD45+ and CD3+ cells, yet its tumors were extensively infiltrated, strongly suggesting clonal proliferation of T cells. The relative distribution of T, B, NK, and myeloid cells within each mouse did not explain the intragroup TIL frequency variation (Fig. 5C), and the applied HSC donors also failed to provide an obvious explanation for the intragroup TIL frequency variation (Fig. 5D).

## 4 Discussion

It is discouraging that 14 of 15 oncology drugs entering phase I trials will eventually fail to be approved by the FDA (Hay *et al*., 2014). A major limitation in rational drug development is the lack of predictive preclinical *in vivo* models. In traditional xenograft models, cancer growth and metastasis occur in the absence of a functional immune system, which may have limited clinical relevance because cancers recruit and interact with various immune cells that are functionally important for the metastatic process. Proper evaluation of novel cancer immune therapeutics requires the coexistence of human cancer and immune cells in an *in vivo* platform in which human cancer growth, metastasis, and treatment occur in the presence of a functional human immune system.

This study describes an advanced mouse model with a HIS and addresses how the immune system interacts with various cancer models, affecting primary tumor growth and metastasis. We found selective accumulation of immune cells at tumor sites, but not in normal tissues. Similar to clinical breast cancers (Savas *et al*., 2016) and consistent with previous reports of triple‐negative breast cancer growth in HIS mice (Wang *et al*., 2018), CD4+ T cells were the major infiltrating TIL subpopulation, with CD8+ T cells accounting for ~ 20–30%. Importantly, there was no correlation between the number of reconstituted immune cells in the blood and within the tumor beds, indicating selection and proliferation of tumor‐reactive immune cells followed by homing to the tumor bed. Furthermore, we found PDL1 upregulation in TIL‐dense areas, and model‐specific accumulation of Tregs demonstrating clinically relevant tumor–immune cell interactions. Taken together, these data strongly indicate ongoing immune reactions toward the cancer transplants that validate the value of the applied models for evaluating immune modulatory anticancer treatment.

Our data demonstrate that BRGS‐HIS mice consistently develop primary tumors that grow close‐to‐synchronous within each model. Previous studies in HSC‐transplanted NSG mice have reported primary tumor development, but with the exception of the study by Wang *et al*. (2018), the growth pattern was either not illustrated or largely heterogeneous (Rongvaux *et al*., 2014; Schilbach *et al*., 2015; Wege *et al*., 2014). The apparent cancer tolerance in BRGS‐HIS mice was not only due to an overwhelming growth rate, since the slow‐growing MDA‐MB‐468 tumors were not rejected. Further, the lack of rejection was not due to the lack of immune system effector functions, as demonstrated by the extensive TIL infiltration, growth inhibition, and increased PDL1 tumor expression in mouse o. Rather, our data suggest a balance between tolerance and immune‐mediated cancer destruction that can be influenced toward the latter when intratumoral CD8+ T cells become sufficiently high. These data, therefore, at least partly recapitulate the clinical situation in which increased infiltration of CD8+ T cells of both melanoma (Clark *et al*., 1989; Clemente *et al*., 1996; Mackensen *et al*., 1993) and triple‐negative breast cancer (Salgado *et al*., 2015; Savas *et al*., 2016) is associated with a more favorable prognosis.

This work demonstrates, for the first time, that metastasis development can be studied in humanized mice. Previously, metastases have only been reported upon ectopic co‐transplantation of human HSC and cancer cells, which may allow cancer cells to spread before the immune system is reconstituted (Wege *et al*., 2014). Moreover, our study is the first to quantify the metastatic burden and correlate it with immune cell infiltration in HIS mice.

Orthotopic transplantation of MDA‐MB‐231 cancer cells in NSG mice has previously been reported to develop consistent lung metastases and occasional liver metastases (Iorns *et al*., 2012). Interestingly, we found (albeit on a very small group size) a strong negative correlation between the extent of CD8+ T cell infiltration in primary MDA‐MB‐231 tumors and their ability to metastasize to the lungs, indicating that CD8+ T cells limit the metastatic capacity. The A375 tumor model is known to be highly metastatic in immune‐deficient models (Lobos‐Gonzalez *et al*., 2014), and interestingly, the tumors were largely immune cell‐excluding in the BRGS‐HIS model, leading to similar metastatic capacity in the two models. As in immune‐deficient mice (Brunner *et al*., 1993), MDA‐MB‐468 tumors exhibited limited growth and metastases in BRGS‐HIS mice. Recently, non‐cell autonomous IL‐11 stimulation was shown to be sufficient to drive MDA‐MB‐468 tumor growth and metastasis in a xenograft model (Marusyk *et al*., 2014). However, in our model, the immune cells either did not provide the necessary IL‐11 stimulation, or the process was counterbalanced by other mechanisms.

Following the recent breakthrough of immune checkpoint blockade, stratifying cancers based on the presence of PDL1 and/or TILs in the tumor and surrounding tumor microenvironment (TME) have been suggested as useful parameters for evaluating the state of the anticancer immune response and thus the potential for therapeutic intervention (Smyth *et al*., 2016; Sznol and Chen, 2013; Taube *et al*., 2012; Teng *et al*., 2015). Because type I TMEs (‘adaptive immune resistance’; PDL1+, TIL+) are thought to respond most effectively to immune checkpoint blockade, treatments that reshape type II (‘immunological ignorance’; PDL−, TIL−), III (‘intrinsic induction’ TIL−, PDL1+), and IV (‘immune tolerance’, PDL−, TIL+) TMEs into a type I TME are actively being pursued. In our hands, the MDA‐MB‐231 model exhibited a typical type I TME with high levels of TILs driving upregulation of PDL1 leading to adaptive resistance. This observation is consistent with the observed growth‐inhibitory effect of PD1 blockade in HIS mice (Wang *et al*., 2018) and indicates that the MDA‐MB‐231 model may prove valuable for evaluating combination partners to PD1/PDL1 blockade, and possibly also treatments directed toward relieving the immune suppressive capacity of human Tregs. It is noteworthy that another group recently reported no effect on MDA‐MB‐231 PDL1 expression when transplanted subcutaneously in HIS mice (Rom‐Jurek *et al*., 2018). Whether this apparent discrepancy is due to differences in transplantation sites or the extent of the immune responses within each mouse warrants further investigations.

The MDA‐MB‐468 model presented with a type IV TME, with apparent immune tolerance and lack of PDL1 expression. It could, therefore, be valuable for targeting or reshaping of type IV to type I TMEs. Interestingly, MDA‐MB‐468 tumors consistently recruited Tregs, which may aid tolerance. Finally, the A375 model presented with an immune cell‐excluding type III TME. Radiation therapy alone or in combination with other interventions has been shown to effectively alleviate T‐cell exclusion in translational models (Klug *et al*., 2013). Since BRGS mice do not carry the Prkdc^*scid*^ mutation, they remain largely radiation‐resistant. We therefore hypothesize that A375 tumors grown in BRGS‐HIS mice may serve as an archetype model system to identify and evaluate therapeutic strategies (including radiation therapy) that reshape type III (and II) to type I TMEs.

We acknowledge that the HIS s are not without shortcomings, including faulty T‐cell selection, HLA mismatch, abnormal lymphoid structures, suboptimal trophic support, and skewed immune cell ratios and absolute frequencies. Notwithstanding these limitations, our data indicate that HIS mice may provide a powerful model in which to evaluate immune‐modifying anticancer treatments. Considering the variations in TIL frequencies within mice transplanted with the same cancer model, we recommend considering the mice as individuals rather than inbred littermates. Thus, future studies may have to include large numbers of mice to provide reliable scientific answers. Although we did not identify any donor bias, it has recently become possible to expand HSC *in vitro*, allowing larger studies from a single immune system donor (Morton *et al*., 2016). We anticipate that immune systems reconstituted from a single donor will provide more homogenous responses.

## 5 Conclusion

In conclusion, our study shows that both ectopic‐ and orthotopic‐transplanted cancers were able to develop primary tumors and form spontaneous metastases in BRGS‐HIS mice. Importantly, the applied *in vivo* tumor models exhibited very diverse interactions with the human immune system, creating characteristic TMEs that exploited immune escape strategies similar to cancers in patients. These models may, therefore, serve as important tools for future preclinical investigations.

## Author contributions

OLG: Conception and design, collection and assembly of data, data analysis and interpretation, manuscript writing, final approval of manuscript. MGT: Conception and design, collection and assembly of data, data analysis and interpretation, manuscript writing, final approval of manuscript. BP: Data analysis and interpretation, final approval of manuscript. HJD: Conception and design, data analysis and interpretation, financial support, manuscript writing, final approval of manuscript.

## Supporting information


**Fig. S1.** Lung and liver macro metastases derived from A375 and MDA‐MB‐231 primary tumors.

**Fig. S2.** Brain metastases did not develop from A375 primary tumors.

**Fig. S3.** Distant metastases developed in both liver and lungs, but not in other organs, from luciferase‐transduced MDA‐MB‐231 primary tumors, while no distant metastases developed from MDA‐MB‐468 primary tumors.

**Fig. S4.** Infiltrating T cells in tumors from mouse o.

**Fig. S5.** Tumors grown in BRGS‐HIS‐mice contained a variety of immune cells.

**Fig. S6.** Tregs did not infiltrate A375 tumors.

**Table S1.** Characteristics of the individual HIS‐mice.
