# Supplementary material for: Human cancer evolution in the context of a human immune system in mice
Source: Mol Oncol. 2018 Sep 3;12(10):1797–810. doi: 10.1002/1878-0261.12374 (PMC6165999; doi:10.1002/1878-0261.12374)
Supplement: Supplementary file 1 — Fig. S1. Lung and liver macro metastases derived from A375 and MDA‐MB‐231 primary tumors. [file MOL2-12-1797-s001.docx]

**
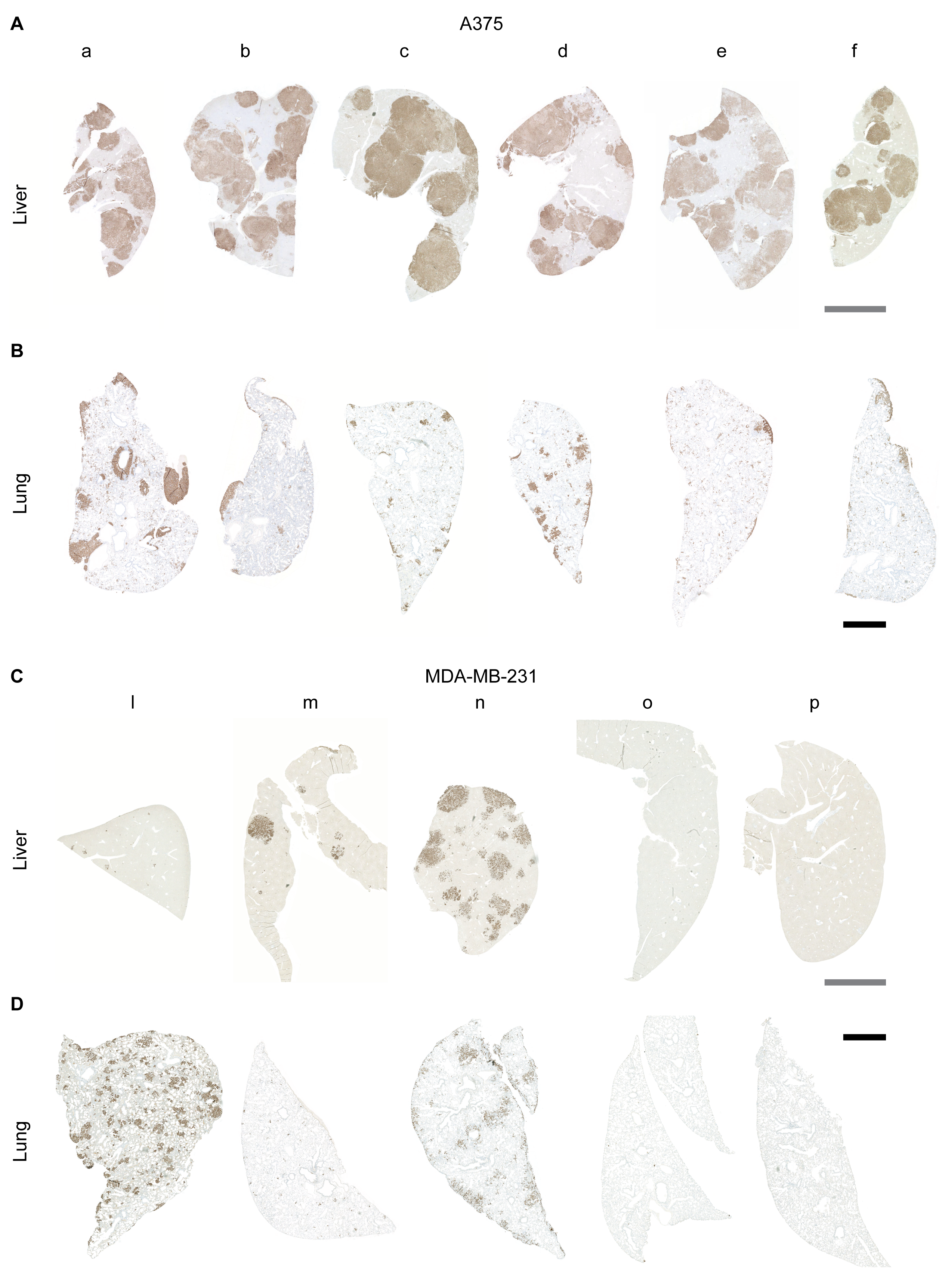
**

**Figure S1. Lung and liver macro metastases derived from A375 and MDA-MB-231 primary tumors.** All A375-challenged mice developed large liver (A) and lung (B) metastases. Only mice l, m, and n developed large MDA-MB-231 liver (**C**) and lung (**D**) metastases. Cancer cells were detected by IHC staining using antibodies against human EGFR (A375) or pan-cytokeratin (MDA-MB-231). Grey and black scale bars: 5 and 1 mm, respectively.
