# Supplementary material for: Human cancer evolution in the context of a human immune system in mice
Source: Mol Oncol. 2018 Sep 3;12(10):1797–810. doi: 10.1002/1878-0261.12374 (PMC6165999; doi:10.1002/1878-0261.12374)
Supplement: Supplementary file 2 — Fig. S2. Brain metastases did not develop from A375 primary tumors. [file MOL2-12-1797-s002.docx]

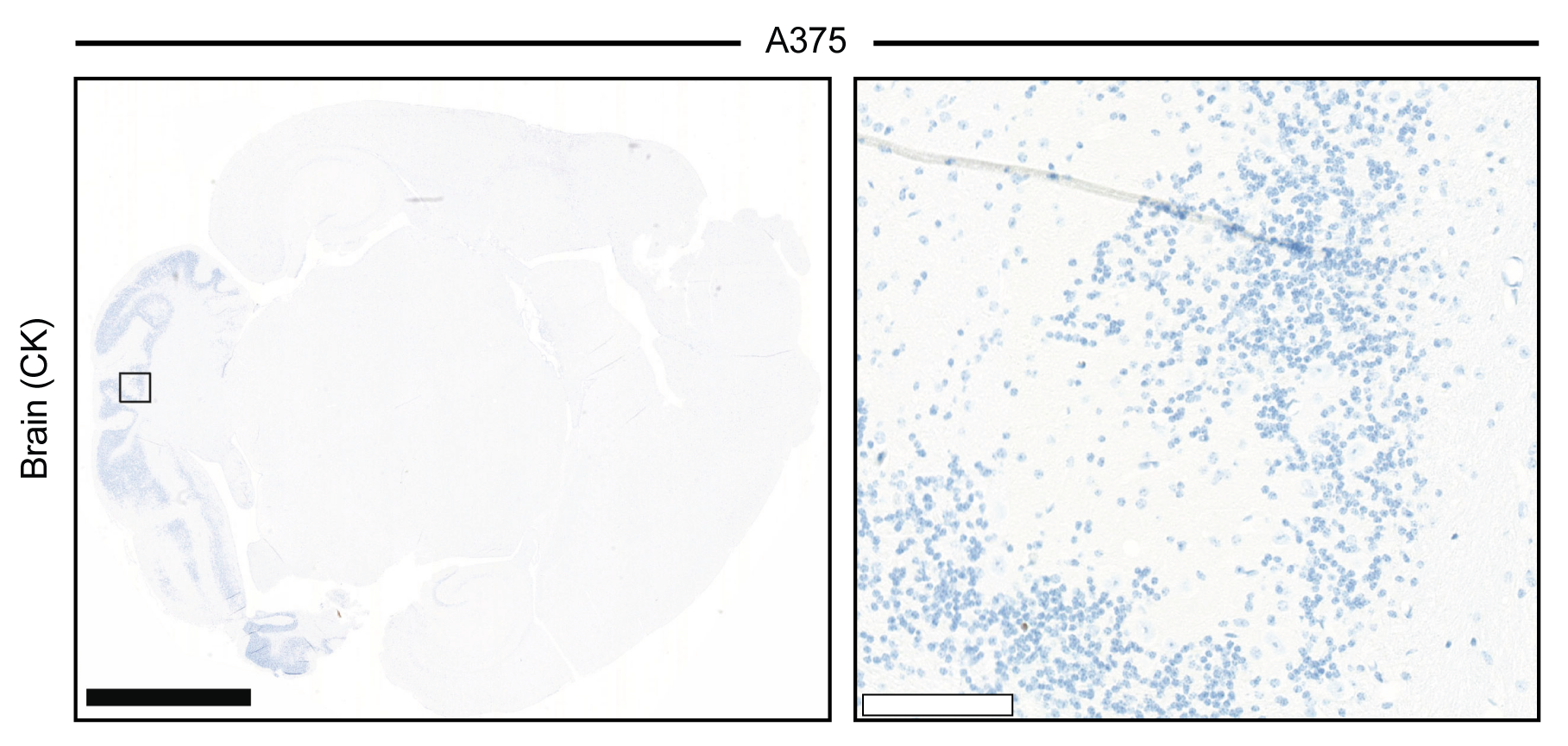


**Figure S2. Brain metastases did not develop from A375 primary tumors**. Metastases in the excited mouse brains were evaluated by immunohistochemical staining using an antibody against human EGFR. Left image is a magnification of boxed area in the right image showing no sign of micro and macro metastases. Black and white scale bars: 2.5 mm and 100 μm, respectively.
