# Supplementary material for: Human cancer evolution in the context of a human immune system in mice
Source: Mol Oncol. 2018 Sep 3;12(10):1797–810. doi: 10.1002/1878-0261.12374 (PMC6165999; doi:10.1002/1878-0261.12374)
Supplement: Supplementary file 3 — Fig. S3. Distant metastases developed in both liver and lungs, but not in other organs, from luciferase‐transduced MDA‐MB‐231 primary tumors, while no distant metastases developed from MDA‐MB‐468 primary tumors. [file MOL2-12-1797-s003.docx]

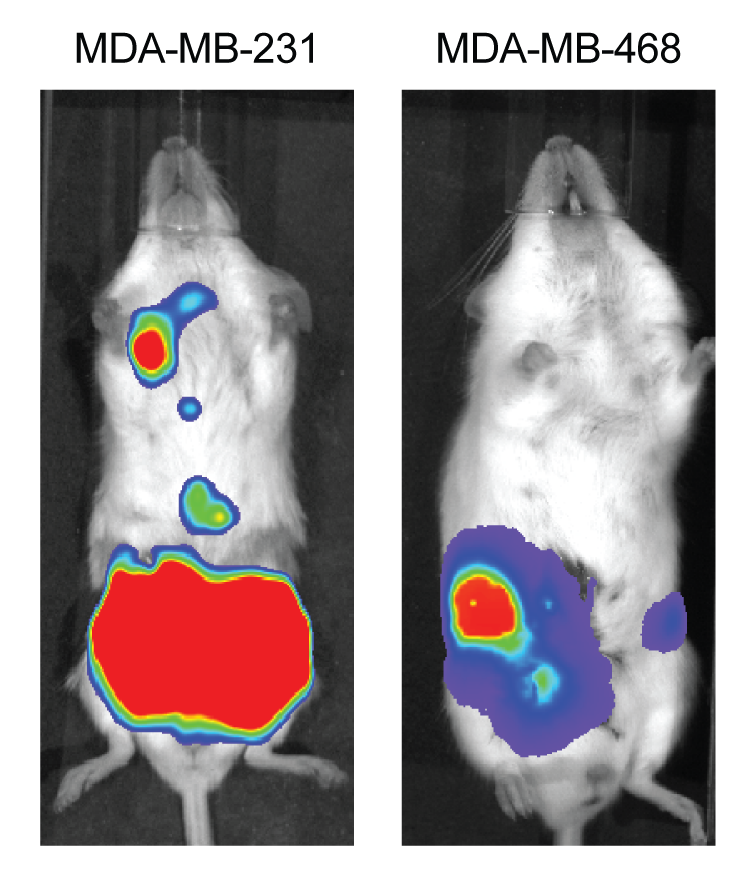


**Figure S3. Distant metastases developed in both liver and lungs, but not in other organs, from luciferase-transduced MDA-MB-231 primary tumors, while no distant metastases developed from MDA-MB-468 primary tumors.** Representative mice are shown.
