# Supplementary material for: Human cancer evolution in the context of a human immune system in mice
Source: Mol Oncol. 2018 Sep 3;12(10):1797–810. doi: 10.1002/1878-0261.12374 (PMC6165999; doi:10.1002/1878-0261.12374)
Supplement: Supplementary file 4 — Fig. S4. Infiltrating T cells in tumors from mouse o. [file MOL2-12-1797-s004.docx]

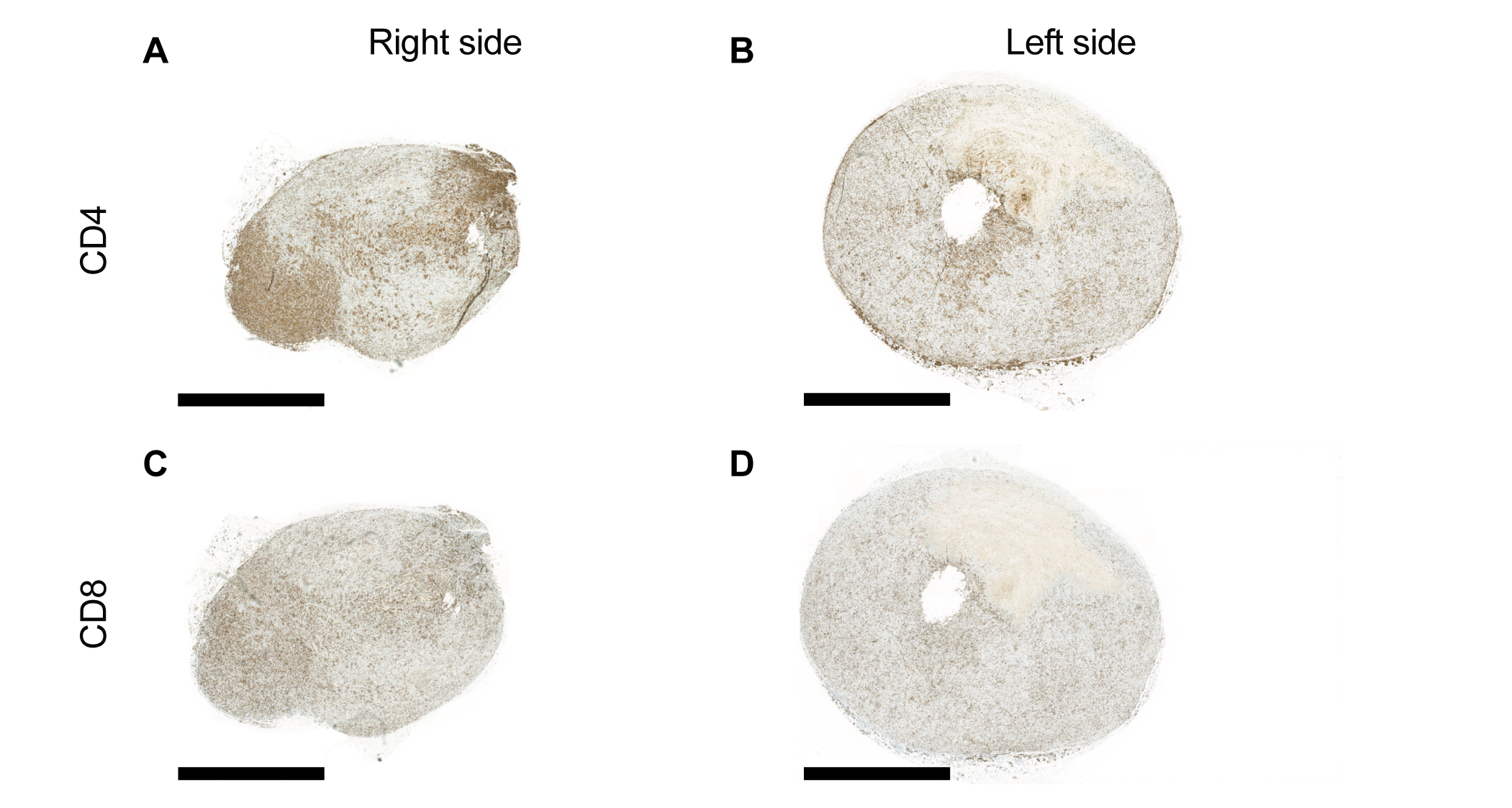


**Figure S4. Infiltrating T cells in tumors from mouse o.** Both CD4+ (**A-B**) and CD8+ (**C-D**) cells extensively infiltrated both left and right-sided MDA-MB-231 tumors in mouse o. Scale bar: 2.5 mm.
