# Supplementary material for: Human cancer evolution in the context of a human immune system in mice
Source: Mol Oncol. 2018 Sep 3;12(10):1797–810. doi: 10.1002/1878-0261.12374 (PMC6165999; doi:10.1002/1878-0261.12374)
Supplement: Supplementary file 5 — Fig. S5. Tumors grown in BRGS‐HIS‐mice contained a variety of immune cells. [file MOL2-12-1797-s005.docx]

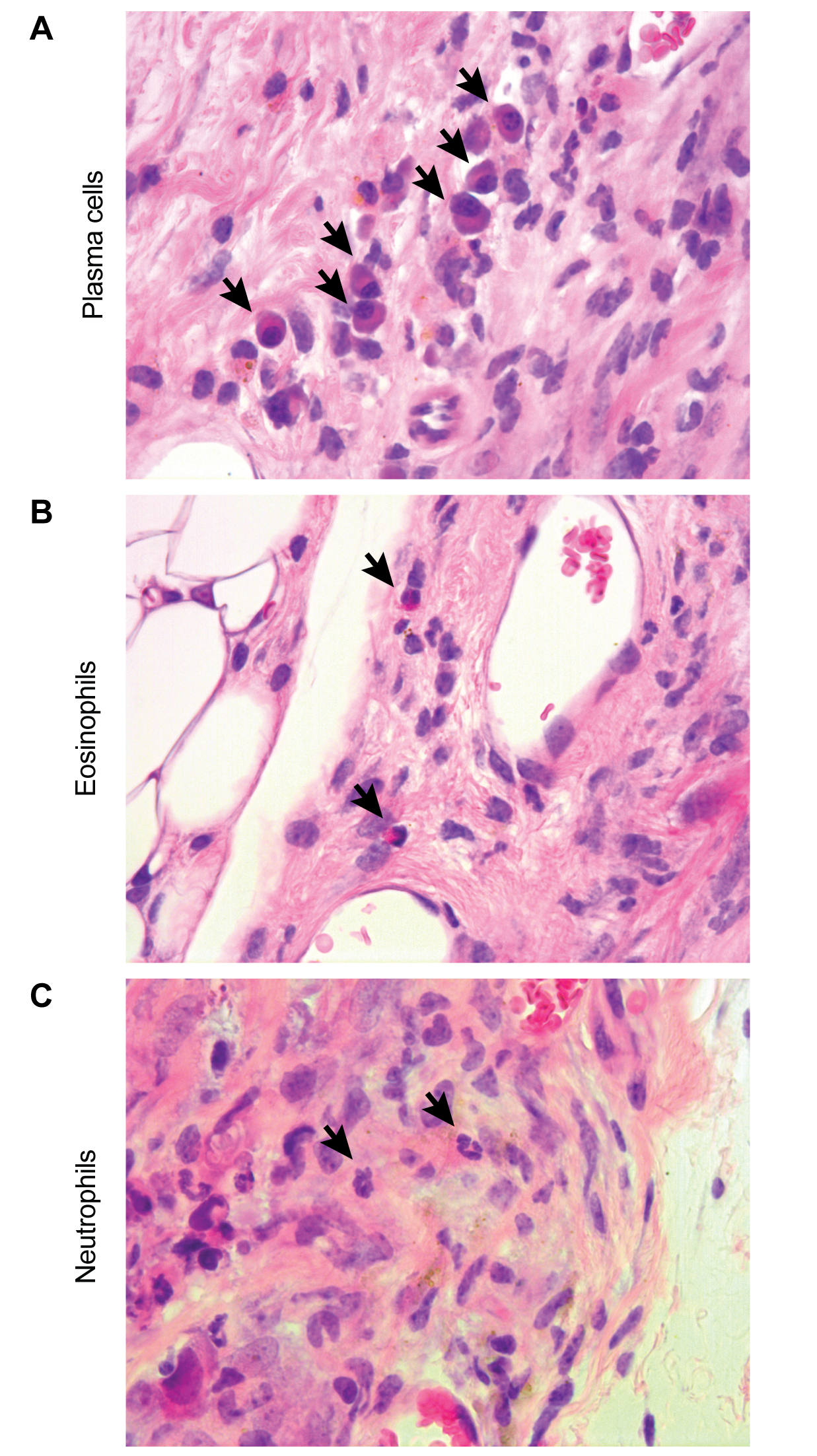


**Figure S5. Tumors grown in BRGS-HIS-mice contained a variety of immune cells.** These immune cells included plasma cells (**A**), eosinophil granulocytes (**B**) and neutrophil granulocytes (**C**)**.** Cells of interest are indicated by arrows.
