# Supplementary material for: Human cancer evolution in the context of a human immune system in mice
Source: Mol Oncol. 2018 Sep 3;12(10):1797–810. doi: 10.1002/1878-0261.12374 (PMC6165999; doi:10.1002/1878-0261.12374)
Supplement: Supplementary file 6 — Fig. S6. Tregs did not infiltrate A375 tumors. [file MOL2-12-1797-s006.docx]

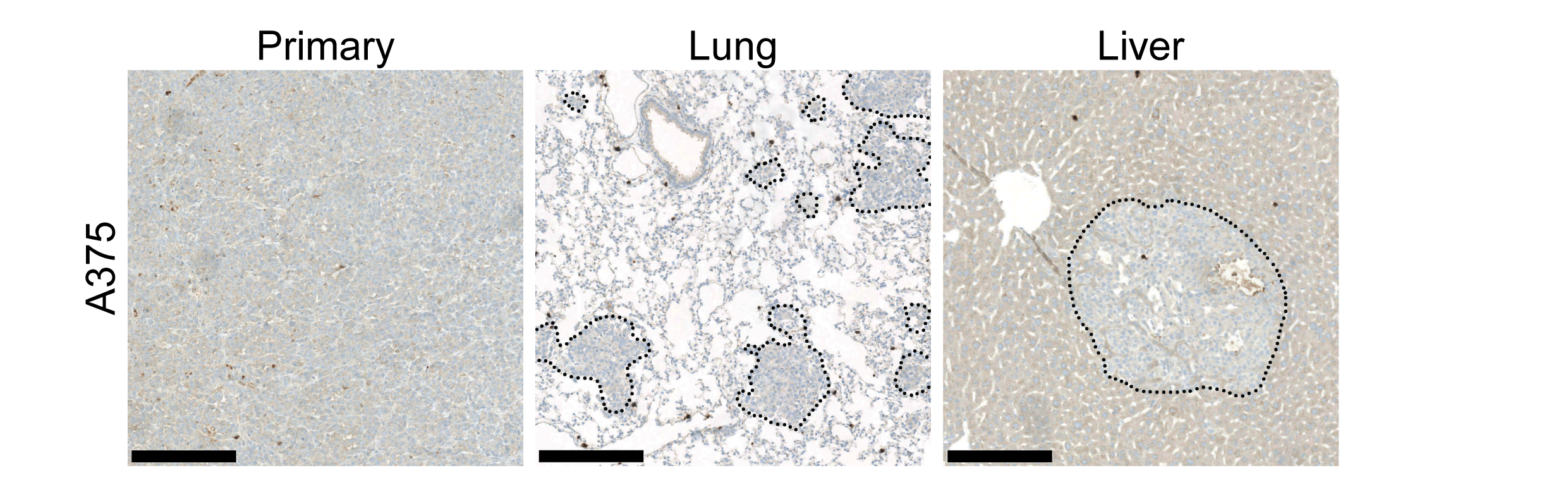


**Figure S6. Regulatory T cells (Tregs) did not infiltrate A375 tumors.** FoxP3+ cells were largely absent in both primary and metastatic lung and liver A375 lesions. Scale bar: 250 μm.
